# Treatment outcome and prognostic factor analysis in transplant-eligible Chinese myeloma patients receiving bortezomib-based induction regimens including the staged approach, PAD or VTD

**DOI:** 10.1186/1756-8722-5-28

**Published:** 2012-06-08

**Authors:** Chor Sang Chim, Albert Kwok Wai Lie, Eric Yuk Tat Chan, Herman Sung Yu Liu, Ching Wa Lau, Sze Fai Yip, Joycelyn Sim, Thomas Shek-Kong Wan, Edmond Shiu-Kwan Ma, Raymond Liang, Eric Tse, Yok-Lam Kwong

**Affiliations:** 1 Department of Medicine, Queen Mary Hospital, University of Hong Kong, Hong Kong, Hong Kong; 2 Department of Pathology, Queen Mary Hospital, University of Hong Kong, Hong Kong, Hong Kong; 3 Department of Medicine, Pamela Youde Eastern Hospital, Hong Kong, Hong Kong; 4 Department of Medicine, Tuen Mun Hospital, Hong Kong, Hong Kong; 5 Department of Medicine, Princess Margaret Hospital, Hong Kong, Hong Kong; 6 Department of Pathology, Hong Kong Sanatorium & Hospital, Hong Kong, Hong Kong

**Keywords:** Myeloma, Staged approach, PAD, VTD, Prognostic factors, Deep vein thrombosis

## Abstract

**Background:**

We have reported promising outcomes using a staged approach, in which bortezomib/thalidomide/dexamethasone was used only in 14 patients with suboptimal response to VAD (vincristine/adriamycin/dexamethasone) before autologous stem cell transplantation (ASCT). Here we compared the outcomes of the staged approach with frontline PAD (bortezomib/doxorubicin/dexamethasone) or VTD (bortezomib/thalidomide/dexamethasone) induction, and analysed prognostic factors for outcome.

**Patients and methods:**

Ninety-one transplant-eligible Chinese patients received three induction regimens prior to ASCT [staged approach (N = 25), PAD (N = 31), VTD (N = 35)]. and received thalidomide maintenance for 2 years post-ASCT.

**Results:**

43 (47.3%) patients had International Staging System (ISS) III disease. By an intention-to-treat analysis, the overall CR/nCR rate were 37.4% post-induction, and 62.6% post-ASCT. Five-year overall (OS) and event-free (EFS) survivals were 66% and 45.1%. There was no difference of the post-induction CR/nCR rate, EFS or OS between patients induced by these three regimens. Moreover, ISS III disease did not affect CR/nCR rates. Multivariate analysis showed that ISS and post-ASCT CR/nCR impacted OS while ISS and post-induction CR/nCR impacted EFS.

**Conclusions:**

These three induction regimens produced comparable and favorable outcomes in myeloma. The unfavorable outcome of ISS stage III persisted despite upfront/early use of bortezomib. CR/nCR predicted favorable survivals.

## Background

Bortezomib, a proteasome inhibitor, is an active agent for the treatment of myeloma. Its efficacy was initially demonstrated in the salvage treatment of refractory myeloma patients, with a complete response (CR) rate of 9% [1,2]. Subsequently, a high CR rate has also been shown with the use of bortezomib-based regimens as induction therapy for newly diagnosed myeloma patients [3]. A post-induction CR rate of 43% and 30% was observed when bortezomib-based induction regimens were used in both transplant-eligible and transplant-ineligible myeloma patients [4,5].

In Hong Kong, we have adopted a staged approach, in which newly diagnosed, transplant-eligible myeloma patients are risk-stratified according to their initial chemosensitivity. Patients who respond to vincristine, adriamycin and dexamethasone (VAD) undergo autologous stem cell transplantation (ASCT). Patients who do not respond optimally to VAD receive salvage therapy with bortezomib/thalidomide/dexamethasone (VTD) before ASCT. This staged approach aims at employing early bortezomib-based therapy in patients who do not achieve a rapid cytoreduction after VAD, thereby restricting the use of the expensive bortezomib to patients with suboptimal response to conventional treatment while ensuring “early” bortezomib-based induction therapy. With this strategy, a CR rate of 48% (by an intention-to-treat analysis), and a 3-year overall survival of 75%, has been achieved [6]. Based on this approach, we showed that only 56% myeloma patients required salvage therapy with VTD. During the same study period, two other bortezomib-containing regimens VTD and PAD (bortezomib, doxorubicin, dexamethasone) were also used as first-line treatment of myeloma patients.

In this report, we examined the hypothesis that myeloma patients treated by the staged approach, in which early bortezomib-based induction is used in selected patients, might achieve similar outcomes as compared with patients receiving receiving frontline bortezomib-based regimens.

## Patients and methods

### Patients

Ninety-one newly diagnosed, symptomatic, transplant-eligible myeloma patients with measurable disease were studied. Patients who were financially competent received frontline therapy with bortezomib-containing regimens. Other patients received a staged approach, and were treated with bortezomib supported by philanthropy only when the initial response was suboptimal.

### Regimens

#### Staged approach

**Figure 1:**
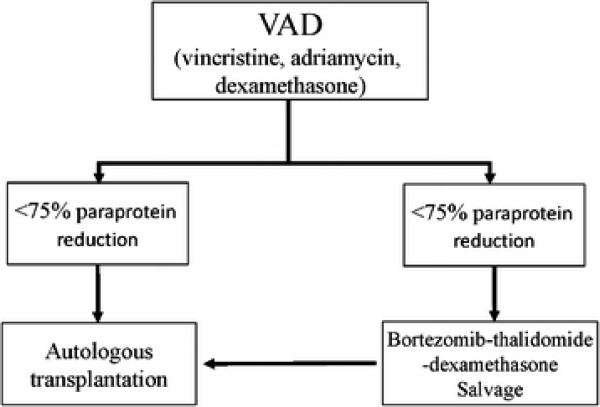
Staged Approach.

Twenty-five patients received initial cytoreduction with three cycles of VAD (vincristine, adriamycin and dexamethasone). (Figure 1) Those achieving ≥ 75% reduction in paraprotein proceeded to ASCT (N = 11; 44%). Patients with < 75% reduction in paraprotein received salvage therapy with four cycles of VTD (bortezomib: 1.3 mg/m^2^/day intravenously on days 1, 4, 8 and 11; thalidomide: 200 mg/day; dexamethasone: 40 mg/d orally from days 1–4 and days 8–11), and then ASCT (N = 14; 56%). All patients received thalidomide (50-100 mg/day) as maintenance therapy post-ASCT.

#### Bortezomib-containing regimens

During the same period, 66 patients received 4 cycles of bortezomib-containing regimens (PAD, N = 31; and VTD, N = 35) followed by ASCT. Patients receiving **PAD** had four 3-weekly cycles of chemotherapy comprising bortezomib 1.3 mg/m^2^/dose on days 1, 4, 8 and 11, adriamycin 9 mg/m^2^/d on days 1–4 and dexamethasone 20 mg/d days 1–4, and days 8–11. Patients receiving **VTD** had four 3-weekly cycles of chemotherapy comprising bortezomib 1.3 mg/m^2^/dose on days 1, 4, 8 and 11, thalidomide 200 mg/d, and dexamethasone 20 mg/d days 1–4, and days 8–11. All patients received maintenance thalidomide (50-100 mg/day) for 2 years post-ASCT.

### Stem cell mobilization and conditioning

Stem cells were mobilized with cyclophosphamide (3 g/m^2^ intravenously) and granulocyte-colony stimulating factor (300 microgram/day subcutaneously until leucocyte recovery). At least 4 x 10^6^ CD34+ cells/Kg recipient body weight were collected. ASCT conditioning regimen comprised intravenous melphalan 200 mg/m^2^.

#### Staging and laboratory investigations

Myeloma work-up included bone marrow examination, skeletal survey, serum β_2_-microglobulin (β_2_M) level, serum protein electrophoresis (SPE), urine protein electrophoresis (UPE), serum or urine immunofixation, paraprotein level assay and serum free light chain (FLC) assay (Freelite, The Binding Site, Birmingham, UK) [7].

#### Fluorescence in-situ hybridization (FISH)

Detection of cytogenetic aberrations was performed on myeloma cells in the bone marrow sample by FISH. Enrichment for myeloma cells was achieved by sorting with CD138 immunomagnetic beads (MiniMACS, Miltenyi Biotec, Auburn, CA) if the percentage of myeloma cells were below 50% of all nucleated cells after haematopathology review. The FISH probes (Abbott Molecular, Abbott Park, IL) comprised the IGH/FGFR3 dual colour dual fusion translocation probe for detection of t(4;14)(p16;q32), the IGH/MAF dual colour dual fusion translocation probe for detection of t(14;16)(q32;q23) and TP53/CEP17 dual colour probe for the detection of p53 deletion, in accordance with the International Myeloma Workshop Consensus recommendation. At least 200 nuclei were analyzed and scored independently by two persons. The cutoff for positivity was above 5% or at least 10 positive nuclei based on test validation data. High-risk (HR) karyotypes include t(4;14), t(14;16) or del(17p).

#### Response criteria

All patients were analyzed on an intention-to-treat basis. Bone marrow plasmacytosis and paraprotein levels were assessed prior to treatment, after VAD, after VTD, and 3 and 6 months after ASCT. Responses were defined according to standard criteria [8]. Complete remission (CR) was defined as complete resolution of disease with absent paraprotein, as evidenced by a negative SPE and immunofixation, and <5% plasma cells in the bone marrow. Near-complete remission (nCR) was defined as a negative SPE but positive immunofixation. Partial response (PR) was subclassified into very good partial remission (VGPR, paraprotein reduction >90%), and PR (paraprotein >50% reduction). Minor response (MR) was defined as paraprotein reduction of >25% but <50%. No response (NR) was defined as paraprotein reduction of <25%. Progression was defined as >25% paraprotein increase in two consecutive tests four weeks apart. Relapse was defined as reappearance of the paraprotein on immunofixation in CR patients, positive SPE in the nCR patients, and/or appearance of new bone lesions. For patients with light chain myeloma, CR was defined as normalization of the level and ratio of serum FLC, and negative serum and urine immunofixation.

### Statistical analysis

OS was defined as time from commencement of induction therapy to death or last follow-up. Event-free survival (EFS) was defined as time from commencement of induction therapy to the date of progression, relapse or death. Patients were clinically staged according to the international staging system (ISS) [9]. Survival curves were plotted by Kaplan-Meier method and compared by the log-rank test. The association of diagnostic clinical parameters (ISS) and treatment response (post-induction CR/nCR or ≥ VGPR, and post-ASCT CR/nCR and ≥ VGPR) with categorical variables including gender, ISS stage, immunoglobulin isotype, post-induction CR/VGPR or post-ASCT CR/VGPR was studied by Chi-Square test, and age by Student’s t-test. Multivariate Cox regression analysis was performed to analyze the impact on OS and EFS of risk factors including pre-treatment clinical characteristics including age, gender, ISS, isotype, and post-treatment responses (post-induction CR/nCR and ≥ VGPR, or post-ASCT CR/nCR and ≥ VGPR) using Statistical Package for the Social Sciences (SPSS) version 16.0. Data on high-risk karyotype was only available in 27 patients, and hence not included in the multivariate analysis. All p-values were two-sided.

## Results

### Patients

There were 50 (54.9%) men and 41 (45.1%) women with a median age of 55 years (range: 33–65 years). Apart from one patient with insufficient data, there were 29 (32.3%) patients with International Staging System (ISS) stage I, 18 (20%) stage II and 43 (47.7%) stage III disease. There were 40 (44%) IgG, 25 (27.5%) light chain, 15 (16.5%) IgA, seven (7.7%) IgD and four (4.4%) non-secretary myeloma cases. Of the 27 patients with karyotypic data, high-risk karyotype occurred in nine (33.3%) [del(17p) in seven and t(4;14) in two]. The demographics and clinical characteristics of patients receiving the staged approach, PAD and VTD were outlined in Table 1. There was no difference in the mean age, and the distribution of gender, ISS, isotype or high-risk karyotypes between these three regimens, thereby permitting a valid comparison of treatment outcomes.

**Table 1:** Patient demographics and treatment outcomes by regimen

	**Staged Approach N = 25**	**PAD N = 31**	**VTD N = 35**	**p-value**
**(A) Demographics**				
Mean age	52.44	53.42	55.71	0.18
Male gender	17 (68 %)	15 (48.4 %)	18 (51.4 %)	0.296
Isotype				0.504
G	12	15	13	
L	6	8	11	
A	4	2	9	
D	2	4	1	
NS	1	2	1	
ISS				0.83
I	7 (28 %)	11 (35.5 %)	11 (32.4 %)	
II	7 (28 %)	5 (16.1 %)	6 (17.6 %)	
III	11 (44 %)	14 (48.4 %)	17 (50 %)	
High-risk Karyotype	3/5 (60 %)	2/6 (33.3 %)	4/12 (25 %)	0.35
**(B) Outcomes**				
CR/nCR post-induction	6 (24 %)	13 (41.9 %)	15 (42.9 %)	0.268
≥VGPR post-induction	14 (56 %)	26 (83.9 %)	26 (74.3 %)	0.064
CR/nCR post-ASCT	15 (62.5 %)	23 (74.2 %)	19 (63.3 %)	0.569
≥VGPR post-ASCT	19 (79.2 %)	29 (93.5 %)	27 (90 %)	0.242
5-year OS	59.80 %	78.70 %	73.80 %	0.661
5-year EFS	36.30 %	52.50 %	69.80 %	0.516

### Treatment results

The post-induction CR/nCR rate were 24%, 41.9% and 42.9% in patients receiving the staged approach, PAD and VTD respectively (p = 0.268). Post-induction ≥ VGPR rates were 56%, 74.2 % and 63.3% in patients receiving the staged approach, PAD and VTD respectively (p = 0.064). While there was a trend of lower ≥ VGPR rate in patients receiving the staged approach compared with frontline PAD or VTD, the difference disappeared after ASCT (post-ASCT ≥ VGPR rate in staged approach, PAD and VTD were 79.2%, 93.5% and 90%; p = 0.242). Of the whole group of 91 patients, the overall CR/nCR rate and ≥ VGPR rates were 37.4% and 72.5% after induction therapy, and 62.6% and 82.4% after ASCT.

### Prognostic indicators

To identify potential risk factors impacting the achievement of CR/nCR or ≥ VGPR either post-induction or post-ASCT, parameters including regimen, isotype, ISS gender or presence of high-risk karyotype were correlated with the achievement of CR/nCR and ≥ VGPR post-induction and post-ASCT (Table 2). Of these factors, induction CR/nCR rate was not impacted by regimen (p = 0.268), ISS (p = 0.781) or gender (p = 0.518) but impacted by isotype (p = 0.017) with lower post-induction CR/nCR rate in those with IgG and IgD isotype. Moreover, IgG isotype had a trend for lower ≥ VGPR rate post-induction. In factors analysed for achievement of post-ASCT responses, IgG isotype and male gender were associated with lower CR/nCR rate but none of these factors correlated with the achievement of ≥ VGPR post-ASCT. Interestingly, neither advanced ISS (ISS III) nor high-risk karyotypes impacted on CR/nCR rates or ≥ VGPR rate after both induction and ASCT.

**Table 2:** Factors impacting post-induction CR/nCR, post-induction VGPR, post-ASCT CR/nCR and post-ASCT VGPR

	**Post-induction CR/nCR (%)**	**Post-induction ≥ VGPR (%)**	**Post-ASCT CR/nCR (%)**	**Post-ASCT ≥ VGPR (%)**
**Regimen**				
Staged approach	6 (24 %)	14 (56 %)	15 (62.5 %)	19 (79.2 %)
PAD	13 (41.9 %)	26 (83.9 %)	23 (74.2 %)	29 (93.5 %)
VTD	15 (42.9 %)	26 (74.3 %)	19 (63.3 %)	27 (90 %)
p-value	0.268	0.064	0.569	0.242
Age*				
p-value	0.547	0.698	0.416	0.730
**Gender**				
male	17 (34 %)	34 (68 %)	27 (57.4 %)	40 (85.1 %)
female	17 (41.45 %)	32 (78 %)	30 (78.9 %)	35 (92.1 %)
p-value	0.518	0.349	0.04	0.501
**ISS**				
I	10 (34.48 %)	19 (65.5 %)	20 (71.4 %)	26 (92.9 %)
II	8 (44.44 %)	14 (77.8 %)	13 (72.2 %)	16 (88.9 %)
III	16 (37.21 %)	33 (73.3 %)	24 (63.2 %)	32 (84.2 %)
p-value	0.781	0.513	0.431	0.559
**Isotype**				
G	8 (20.0 %)	23 (57.5 %)	18 (47.4 %)	33 (86.8 %)
L	14 (56.0 %)	20 (80 %)	18 (81.8 %)	19 (86.4 %)
A	7 (46.67 %)	13 (86.7 %)	11 (78.6 %)	13 (92.9 %)
D	2 (28.57 %)	6 (85.7 %)	6 (85.7 %)	6 (85.7 %)
NS	3 (75.0 %)	4 (100 %)	4 (100 %)	4 (100 %)
p-value	0.017	0.065	0.013	0.908

*: p-value refers to the comparison of the mean age of patients achieving or not achieving a response(CR/nCR or ≥ VGPR).

### Survivals

**Figure 2:**
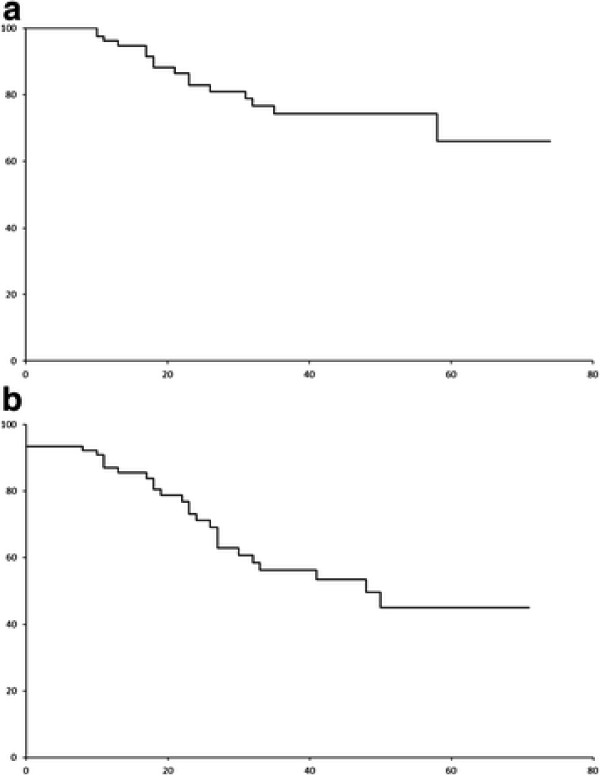
(A) OS of combined cohort of 91 patients; (B) EFS of combined cohort of 91 patients.

**Figure 3:**
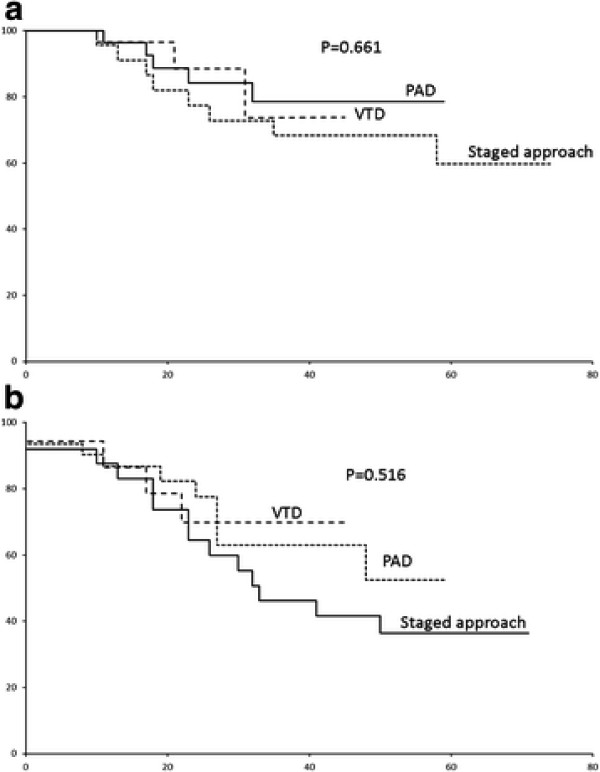
(A) OS by regimens (staged approach, PAD,VTD); (B) EFS by regimens (staged approach , PAD, VTD).

**Figure 4:**
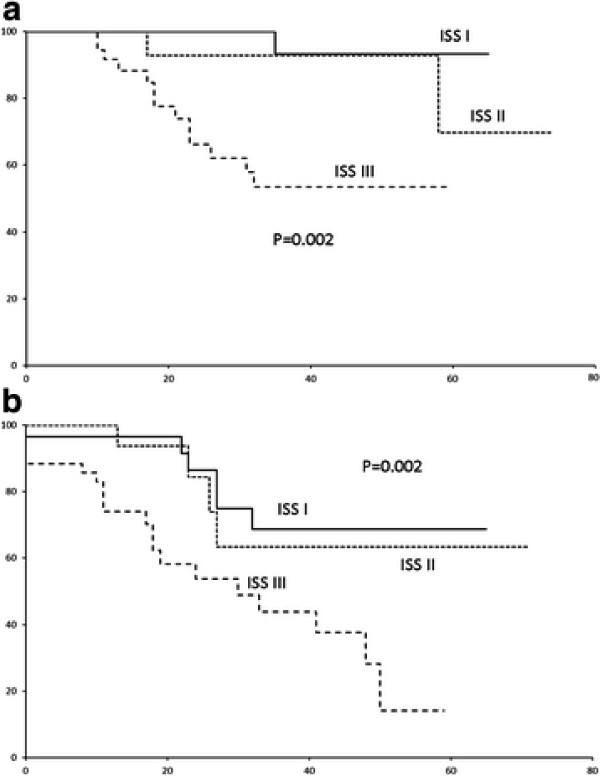
(A) OS by ISS; (B) EFS by ISS.

**Figure 5:**
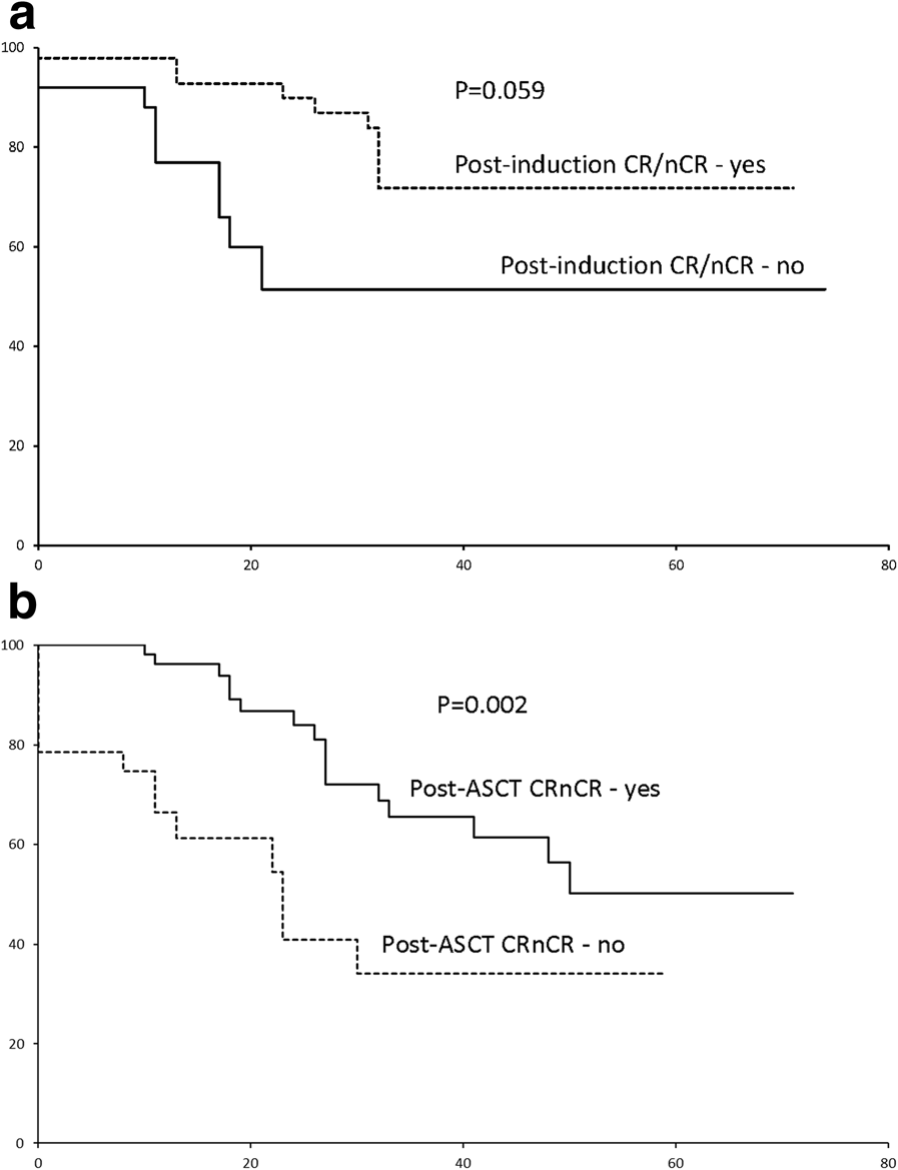
(A) EFS by post induction CR/nCR; (B) OS of post-ASCT/nCR.

In this combined cohort, the 5-year OS and event-free (EFS) survivals were 66% and 45.1% (median EFS: 48 months) (Figure 2A &2B). There was no difference between the induction CR/nCR rate (p = 0.268), EFS (0.516) or OS (p = 0.661) of patients induced by these three regimens (Figure 3 &3B). In univariate analysis, ISS III (Figure 4A), male gender and failure of CR post-ASCT (Figure 5A) predicted an inferior OS (Table 3). On the other hand, ISS III (Figure 4B), failure of CR post-induction (Figure 5B) and failure of CR post-ASCT predicted inferior EFS. In multivariate analysis, advanced ISS and failure of post-ASCT CR/nCR predicted an inferior OS, whereas advanced ISS and failure of CR/nCR post-induction predicted a inferior EFS (Table 4).

**Table 3:** Prognostic factors for overall survival (OS)

	**Univariate analysis**	**Multivariate analysis**		
	**p-value**	**p-value**	**Hazard Ratio**	**95% Confidence Interval**
regimen	0.661	0.320	0.622	0.245 - 1.584
gender	0.043	0.439	0.558	0.128 - 2.443
age	0.256	0.056	1.115	0.997 - 1.248
isotype	0.264	0.160	1.558	0.840 - 2.891
ISS	0.002	0.006	4.323	1.508 - 12.397
Post-induction CR/nCR	0.059	0.291	0.395	0.070 - 2.216
post-induction ≥ VGPR	0.220	0.480	1.922	0.314 - 11.772
Post-ASCT CR/nCR	0.004	0.045	0.138	0.020 - 0.958
Post-ASCT VGPR	0.001	0.836	1.278	0.124 - 13.153

**Table 4:** Prognostic factors for event-free survival (EFS)

	**Univariate analysis**	**Multivariate analysis**		
	**p-value**	**p-value**	**Hazard Ratio**	**95 % Confidence Interval**
regimen	0.516	0.751	0.907	0.495 - 1.661
gender	0.061	0.372	0.640	0.241 - 1.705
age	0.256	0.896	0.995	0.923 - 1.073
isotype	0.237	0.125	1.339	0.922 - 1.944
ISS	0.003	0.022	1.853	1.092 - 3.143
Post-induction CR/nCR	0.010	0.041	0.307	0.099 - 0.953
Post-induction VGPR	0.077	0.313	1.976	0.526 - 7.424
Post-ASCT CR/nCR	0.002	0.205	0.439	0.123 - 1.567
Post-ACST VGPR	<0.001	0.116	0.275	0.055 - 1.374

## Discussion

There are several observations from the study. Firstly, despite this being a retrospective study, we showed that there was no difference in EFS or OS in patients receiving the staged approach and those receiving frontline PAD or VTD, suggesting that the staged approach yielded comparable survivals as patients receiving frontline bortezomib. This is encouraging because only half [14 (56%)] of the patients receiving the staged approach required salvage VTD, and hence the staged approach could be used as a cost-effective but effective regimen in less affluent countries, where frontline bortezomib might not be affordable for the majority of the population. However, while not reaching statistical significance due to the small number of patients, it is notable that the CR/nCR rate of patients receiving frontline bortezomib-based induction was much higher than that of those induced with the staged approach (42.9% versus 24%). Moreover, the 5-year EFS of patients receiving frontline bortezomib-based induction was almost double that of those induced with the staged approach (69.8% versus 36.3%). Similarly, the 5-year OS of patients receiving frontline bortezomib-based induction was much higher than that of those induced with the staged approach (73.8% versus 59.8%). Therefore, whether significant survival difference may emerge between the staged approach and PAD/VTD after prolonged follow-up remains to be seen. Interestingly, while there was a trend of lower ≥ VGPR rate compared to patients receiving PAD or VTD, the difference was abolished after ASCT.

Secondly, a high post-induction CR/nCR (PAD: 41.9%; VTD: 42.9%) and ≥ VGPR rates (PAD: 74.3%; VTD: 83.9%) were observed in patients receiving frontline PAD or VTD induction. These post-induction CR/nCR rates were comparable to other phase II studies, which also reported a high post-induction CR/nCR rate after PAD (29%) or CyBorD (46%) [4,10].

Thirdly, we attempted to analyse prognostic factors impacting outcome in this combined cohort of myeloma patients receiving either *frontline* (PAD/VTD induction) or *early* bortezomib-based induction (in the staged approach). We showed that ISS III remained an important adverse risk factor predicting both inferior OS and EFS, despite patients receiving early or frontline bortezomib-based induction regimens followed by ASCT. However, there was no association between ISS III and presentation parameters including age, gender, isotype or even high-risk karyotypes. Moreover, ISS III did not result in an inferior CR/nCR or ≥ VGPR rate either post-induction or post-ASCT. Therefore, ISS III is a factor that predicted residual chemo-refractory disease, thereby leading to subsequent fatal relapse or disease progression. Indeed, the adverse prognostic impact of advanced ISS stage for progression-free survival has also been demonstrated in phase III studies [11,12]. Importantly, ways to improve the survivals of ISS III myeloma patients are urgently needed. In this connection, new targeted agents or next generation immunomodulatory agents are urgently required [13]. This is particularly important in this era of targeted therapy when the adverse prognostic impact of high-risk karyotypes were shown to be at least partially, if not totally, overcome by the use of bortezomib-based induction regimens. [14] The persistence of the unfavorable prognostic impact of advanced ISS despite bortezomib-based induction poses an important therapeutic challenge.

In addition, we showed that post-induction CR/nCR predicted a superior EFS, and CR/nCR post-ASCT a superior OS. This is consistent with recent studies that CR/nCR or VGPR prior to ASCT is a favorable factor predicting superior progression-free survival [11,12,15], and CR/nCR post-ACST predicts superior OS [16].

In conclusion, a high CR/nCR and VGPR rates were achieved in patients receiving frontline or early bortezomib-based induction therapy, which was associated with favorable OS and EFS. There was no difference between the outcomes of the staged approach and frontline bortezomib-based induction regimens as PAD or VTD. ISS stage III remains an adverse prognostic factor for both EFS and OS despite frontline or early bortezomib-induction, and poses a therapeutic challenge in this era of targeted therapy when the use of bortezomib-based induction has at least partially abolished the unfavorable impact of high-risk karyotypes. CR/nCR post-induction and post-ASCT are favorable factors predicting favorable OS and EFS, and hence are important end-points of therapy.

## Competing interests

All authors report no relevant conflicts of interest.

## Authors' contributions

CSC, AKWL,YLK designed the clinical protocol; CSC, AKWL, HL, CSL, SFY, JS, RL, ET were involved in treating patients and collecting data; EYTC, TSW, ESKM were involved with labroatory diagnosis and FISH studies; CSC, YLK wrote the paper with contributions from the other authors. All authors read and approved the final manuscript.
